# Bacterial extracellular vesicles in the initiation, progression and treatment of atherosclerosis

**DOI:** 10.1080/19490976.2025.2452229

**Published:** 2025-01-22

**Authors:** Yuling Lin, Jingyu Wang, Fan Bu, Ruyi Zhang, Junhui Wang, Yubing Wang, Mei Huang, Yiyi Huang, Lei Zheng, Qian Wang, Xiumei Hu

**Affiliations:** a Department of Laboratory Medicine, Nanfang Hospital, Southern Medical University, Guangzhou, China; b Center for Clinical Laboratory, Zhujiang Hospital, Southern Medical University, Guangzhou, China; c Institute of Hematology, Zhejiang Engineering Laboratory for Stem Cell and Immunotherapy, Zhejiang University, Hangzhou, China

**Keywords:** Bacterial extracellular vesicles, atherosclerosis, outer membrane vesicles

## Abstract

Atherosclerosis is the primary cause of cardiovascular and cerebrovascular diseases. However, current anti-atherosclerosis drugs have shown conflicting therapeutic outcomes, thereby spurring the search for novel and effective treatments. Recent research indicates the crucial involvement of oral and gastrointestinal microbiota in atherosclerosis. While gut microbiota metabolites, such as choline derivatives, have been extensively studied and reviewed, emerging evidence suggests that bacterial extracellular vesicles (BEVs), which are membrane-derived lipid bilayers secreted by bacteria, also play a significant role in this process. However, the role of BEVs in host-microbiota interactions remains insufficiently explored. This review aims to elucidate the complex communication mediated by BEVs along the gut-heart axis. In this review, we summarize current knowledge on BEVs, with a specific focus on how pathogen-derived BEVs contribute to the promotion of atherosclerosis, as well as how BEVs from gut symbionts and probiotics may mitigate its progression. We also explore the potential and challenges associated with engineered BEVs in the prevention and treatment of atherosclerosis. Finally, we discuss the benefits and challenges of using BEVs in atherosclerosis diagnosis and treatment, and propose future research directions to address these issues.

## 1. Introduction

Atherosclerosis is a progressive inflammatory disease with metabolic disruption. It is typically initiated by endothelial damage and activation, which facilitates the accumulation of lipids and monocytes in the intima of medium- to large-sized arteries. Monocytes differentiate into macrophages, which engulf oxidized low-density lipoproteins to form foam cells. This intricate and chronic inflammatory process, along with impaired clearance functions of macrophages, promotes calcium mineral accumulation and lipid necrotic core formation. Over time, these changes increase the risk of plaque rupture and thrombosis, potentially leading to severe outcomes like myocardial infarction and stroke.^1,2^ The pathogenesis of arteriosclerosis is complex. Nearly 50% of patients with Atherosclerotic Cardiovascular Disease (ASCVD) exhibit few or no traditional risk factors, such as dyslipidemia, hypertension, and diabetes,^3^ suggesting the presence of unknown risk factors that drive disease onset and progression. Current treatments for ASCVD focus on statins to lower serum cholesterol, platelet aggregation inhibitors, and surgical interventions.^4^ While these strategies effectively reduce cardiovascular mortality, their overall benefits for treating ASCVD have plateaued due to unresolved inflammation, leaving a persistent residual risk of cardiovascular events in these patients.^1,5^ Therefore, developing novel therapeutic strategies for arteriosclerosis remains a critical challenge.

Emerging high-throughput technologies and multi-omics analyses, such as metabolomics and transcriptomics, have shed light on the relationship between the human microbiome and ASCVD.^6,7^ Numerous epidemiological, basic science and clinical studies support a causal link between chronic infection with harmful pathogens and the development of atherosclerosis and its complications.^8–10^ Conversely, other studies highlight the favorable effects of specific gut microbes, probiotics, and their metabolites on ASCVD.^11–13^ The mechanisms underlying the complex and dynamic interactions between the human microbiota and the host primarily involve an intricate network of metabolic pathways and signaling mediated by bioactive molecules. Some of these bioactive molecules are packaged into nanoparticles called extracellular vesicles (EVs). EVs are membrane-bound particles secreted by a wide range of cell types across various biological domains, including eukaryotes, prokaryotes, and archaea.^14^ EVs originating from microbes are crucial mediators of transkingdom communication between microorganisms and their hosts. Initially, they were identified as originating from regulatory vesicle-like structures in the outer membrane of Gram-negative bacteria, known as outer membrane vesicles (OMVs).^15^ Later, it was demonstrated that Gram-positive bacteria are also capable of producing membrane vesicles.^16^ Regardless of their origin, we refer to these bacteria-derived vesicles as BEVs. These BEVs range from 20 to 400 nanometers in diameter and encapsulate a diverse set of bioactive molecules, including lipopolysaccharide (LPS), periplasmic and membrane-bound proteins, peptidoglycans, DNA, RNA, and other compounds.^17^ Shielded from enzymatic degradation by the lipid bilayer membranes, these biomolecules can preserve their stability in the harsh extracellular environment of the digestive tract.^18^

**Figure 1.**
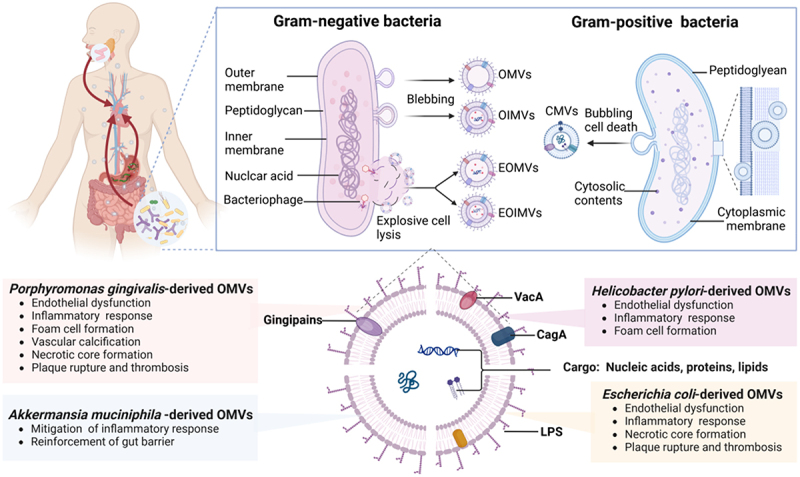
Schematic representation of the biogenesis, circulatory distribution, and impact of BEVs on atherosclerosis.

In recent years, there has been growing interest in the complex relationship between the microbiome and ASCVD. Most research has focused on the impact of diet-dependent metabolites from the gut microbiota on the cardiovascular system (e.g., short-chain fatty acids, trimethylamine-N-oxide, bile acids),^7,19^ while relatively few studies have explored the potential mechanisms by which EVs exert their effects, the microbial metabolites themselves. However, available data confirms that the influence of microbes on the cardiovascular system is at least partially mediated by their EVs. In this review, we will summarize the origins, circulation, and distribution pathways of BEVs, and highlight their multifaceted roles in the pathogenesis, treatment, and prevention of atherosclerosis. Finally, we will discuss the current limitations of BEV research and propose potential future research. Figure 1 provides a concise overview of the biogenesis and circulatory distribution of BEVs, along with the mechanisms by which the BEVs from key atherosclerosis-associated microbiota, as discussed in this paper, contribute to either the progression or attenuation of atherosclerosis.

Gram-negative bacteria generate EVs through membrane blebbing and cell lysis. Blebbing is responsible for the release of OMVs and OIMVs, while explosive cell lysis triggers the formation of EOMVs and EOIMVs. OMVs are abundant in outer membrane components and lack cytoplasmic constituents. However, the weakening of the peptidoglycan layer causes the inner membrane to protrude, leading to the formation of OIMVs. EOMVs and EOIMVs, derived from phage-mediated degradation of the peptidoglycan layer, randomly incorporate cytosolic components. CMVs are generated through “bubbling cell death,” in which endolysins weaken the peptidoglycan layer of Gram-positive bacteria, enabling CMVs to penetrate this structure. After being secreted from their original colonization sites, bacterial EVs can enter the circulation and distribute throughout various organs, including the aorta and heart. Atherosclerosis-related pathogenic and beneficial bacteria carry specific bioactive molecules that either accelerate or inhibit the progression of atherosclerosis by influencing a series of events.

Abbreviations (OMVs: outer membrane vesicles, OIMVs: outer-inner membrane vesicles, EOMVs: explosive outer membrane vesicles, EOIMVs: explosive outer-inner membrane vesicles, CMVs: cytoplasmic membrane vesicles).

## 2. Overview of bacterial extracellular vesicles

### 2.1. Biogenesis and components

The distinct surface compositions of Gram-negative and Gram-positive bacterial cells lead to differences in the biological origins and cargo of their BEVs. Gram-negative bacteria produce EVs through both membrane blebbing and explosive cell lysis.^20^ OMVs that originate from blebbing of the outer membrane are primarily composed of outer membrane and periplasmic components, such as LPS and peptidoglycan. Similarly, outer-inner membrane vesicles (OIMVs), which are formed by the blebbing mechanism involving autolysin-mediated weakening of the peptidoglycan layer, contain both inner and outer membranes of the parent cell, as well as cytoplasmic components.^21^ Phage-derived endolysins degrade the bacterial peptidoglycan layer leading to explosive cell lysis, the resultant explosive outer membrane vesicles (EOMVs) and explosive outer-inner membrane vesicles (EOIMVs) also randomly encapsulate cytoplasmic contents. These subtypes are often collectively referred to as OMVs due to the difficulty in accurate differentiation.^20^ Gram-positive bacteria produce cytoplasmic membrane vesicles (CMVs), which lack an outer membrane. Their production mechanism is partially similar to the explosive cell death observed in Gram-negative bacteria. This process often results in cell death due to the loss of cytoplasmic membrane integrity and is therefore termed “bubbling cell death”.^22^

BEVs harbor a variety of microbe-associated molecular patterns. Notably, OMVs from Gram-negative bacteria are enriched with LPS, whose lipid A moiety is recognized by Toll-like receptor 4 (TLR4). In contrast, the surface of cytoplasmic membrane vesicles from Gram-positive bacteria contains lipoteichoic acid, which is a ligand for TLR2.^17,23^ Pathogenic strains produce BEVs with unique virulence factors, such as cytotoxin-associated gene A (CagA) and vacuolating cytotoxin A (VacA) from *Helicobacter pylori*, and gingipains from *Porphyromonas gingivalis*. These BEVs deliver virulence factors to host cells and contribute to antibiotic resistance and biofilm formation, underscoring their importance in bacterial pathogenesis.^24,25^ Notably, BEV populations are highly complex and heterogeneous. This heterogeneity in molecular composition and size is influenced not only by the parent bacterial strain and biogenesis pathways but also by environmental factors such as pH, temperature, nutrients, and antibiotic exposure, all of which can alter OMVs production, the enrichment of bioactive molecules, and their biological efficacy.^26–28^ Thus, investigating the intricate variations in BEV populations across various physiological and pathological conditions is crucial for conducting reproducible analyses of BEVs and for achieving a comprehensive understanding of their functions within the host.

### 2.2. Translocation and circulation

Various internal and external factors that lead to barrier disruption can significantly increase the entry of BEVs into the systemic circulation.^29^ The primary theoretical basis for the translocation of gut-derived BEVs into the systemic circulation is the increased paracellular permeability due to epithelial barrier disruption, commonly known as “leaky gut”. However, recent studies have indicated that BEVs’ translocation is not confined to conditions of epithelial barrier impairment. BEVs from commensal bacteria can traverse the intestinal epithelium via paracellular pathways and reach systemic organs even under healthy conditions.^30^ Furthermore, research has found that BEVs are present in the blood not only of patients with gastrointestinal diseases but also of healthy blood donors, with the majority originating from the gut microbiota.^31,32^ Fluorescently labeled OMVs exhibit rapid traversal of the gastrointestinal mucosal barrier after gastric gavage and extensive dissemination to distant organs, maintaining persistence in mice for several days.^33,34^ The mechanisms by which BEVs traverse an intact epithelial barrier and enter the bloodstream under healthy conditions remain to be fully elucidated. Several studies have summarized the most plausible pathways involved, including transcellular migration, paracellular pathways, and direct uptake by dendritic cells.^35,36^ In addition to systemic dissemination via the bloodstream, BEVs can also be detected in the trigeminal ganglion and hippocampus following gingival exposure to *Porphyromonas gingivalis* extracellular vesicles. This finding suggests that the nucleic acids and other bacterial products detected in the brains of Alzheimer’s disease patients may be due to the transfer of EVs (rather than *P. gingivalis*) from the gingiva to the brain via the trigeminal nerve.^37^ These studies indicate that BEVs represent a sophisticated bacterial strategy to exert effects in distant organs beyond the originally colonized site.

### 2.3. Biological effects and biomedical potential

The bioactive components released via BEVs not only have a greater chance of avoiding degradation and thus being transported over long distances, but they also be concentrated, allowing a single vesicle to deliver a sufficient quantity of molecules directly to the target cell, ensuring their biological activity.^20^ In a study investigating the gut microbiota and osteoarthritis, while comparable amounts of the purified recombinant pathogenic factor could induce inflammation in vitro, it failed to replicate the same exacerbation of arthritis observed with OMV-carried pathogenic molecules in vivo. This finding underscores the critical role of BEVs as transport vehicles for specific bioactive molecules, enabling their action at distant sites.^38^ BEVs interact with host cells through various mechanisms, including direct activation of surface receptors via ligand binding and delivery of effector molecules to the cytoplasm through direct membrane fusion or uptake by recipient cells via various endocytic pathways such as macropinocytosis, phagocytosis, and endocytosis.^39,40^ The magnitude of the biological effects of BEVs on the host may depend on the type, dose, distribution kinetics, and half-life of the BEV populations. It has been reported that the concentration of BEVs in the plasma of non-septic patients ranges between 10^5 and 10^6/mL, and their quantity is associated with increased permeability of the intestinal epithelial barrier.^41^ Once BEVs enter the systemic circulation, they may be cleared in highly perfused areas such as the liver, affected by their origin, concentration, and composition (e.g., the presence of specific membrane proteins targeting clearance organs like the liver). Much remains to be explored regarding their physical excretion.

Pathogenic gut microbial BEVs can induce damage in distant organs and tissues, such as osteoarthritis and cognitive impairment.^38,42^ In contrast to pathogenic BEVs that induce host inflammation and pathological damage, BEVs derived from gut commensal bacteria and probiotics may exert beneficial effects on the host by influencing epithelial and immune cell responses, thereby maintaining microbial and gastrointestinal homeostasis.^43,44^ Additionally, BEVs exhibit tremendous potential in biomedical applications due to their inherent structural stability, innate transport capabilities, and ability to overcome biological barriers that traditional synthetic drug carriers, such as liposomes, cannot replicate in cell-cell interactions.^45^ By artificially loading proteins, nucleic acids, and small-molecule drugs, coupled with targeted modification strategies, BEVs can efficiently deliver therapeutics to specific sites, thereby minimizing adverse effects and enhancing therapeutic efficacy.^46^ Moreover, BEVs contain immunogenic proteins from their parent bacteria, which can be effectively recognized and phagocytosed by antigen-presenting cells, establishing them as an ideal source for the design of vaccines and adjuvants.^47^

## 3. Bacterial extracellular vesicles promote atherosclerosis

**Figure 2.**
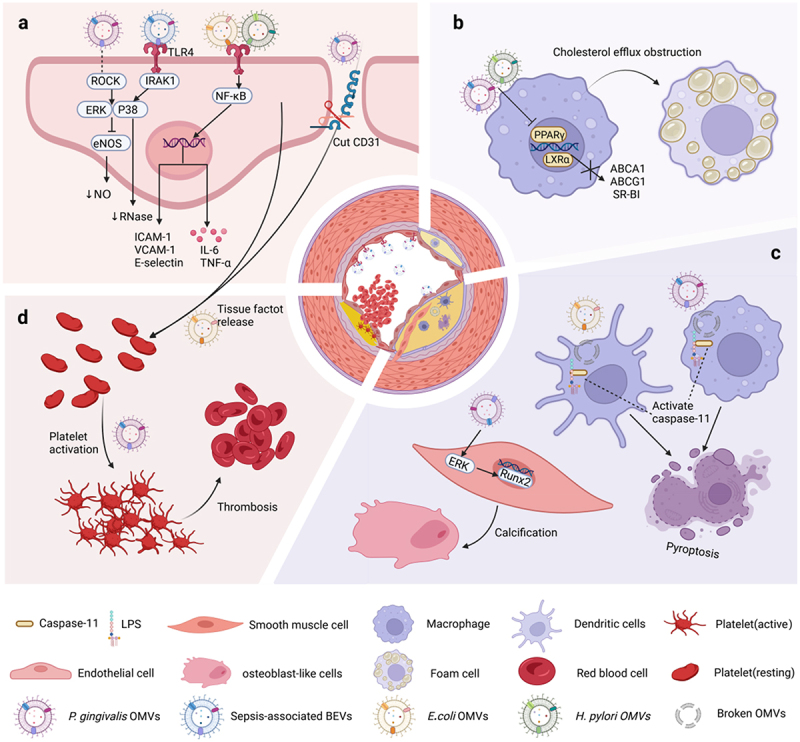
Mechanism of influence of specific bacterial extracellular vesicles on the occurrence and development of atherosclerosis.

As a complicated progressive disease, atherosclerosis primarily consists of several events, such as endothelial cell dysfunction, proinflammatory cytokine secretion, foam cell formation, vascular smooth muscle cell (VSMCs) migration, and abundant cell death. Here, we classify the pathological progression of atherosclerosis into four stages: initiation, onset, progression, and rupture stage (Figure 2), and the major contributions of several BEVs in different stages of atherosclerosis are summarized in Table 1.

**Table 1.** Summary of atherogenic effects induced by bacterial extracellular vesicles.

Stage	Event	Parent bacteria of BEVs	Stimulus component	Mechanism	Model
Initiation	Endothelial dysfunction	Helicobacter pylori	CagA and LPS	Activation of ROS/NF-κB signaling pathway	ApoE–/– male mice and HUVECs
		Porphyromonas gingivalis	Gingipain	Cleavage of CD31	Human microvascular endothelial cells, Zebrafish Larvae Infection Model
		Porphyromonas gingivalis	unknow	Suppressed eNOS expression via activation of the ERK1/2 and p38 MAPK signaling pathways in a Rho kinase-dependent manner.	HUVECs and mouse aorta endothelium
		Klebsiella pneumoniae	unknow	↓ SIRT1 and p-eNOS, ↑ NOX2, COX-2, ET-1 and p53 in endothelial cells	C57BL/6 mice, mouse aortic rings HUVECs
	Imbalance of inflammatory mediators in vascular endothelium	Escherichia coli	unknow	↑ IL-6 by triggering the translocation of NF-κB to the nucleus	HUVECs
		Sepsis-associated bacteria	LPS	Repress endothelial RNase1 via an LPS-induced TLR4-IRAK-1 and p38-mediated mechanism	Human microvascular lung endothelial cells
	Endothelial activation and monocyte recruitment	Escherichia coli	LPS	↑ ICAM-1, E-selectin, or VCAM-1 via the activation of NF-κB.	HUVECs
		Porphyromonas gingivalis	unknow	↑ Chemoattractant proteins including CXCL1, CXCL2, and CXCL8, and E-selectin	HUVECs
		Staphylococcus aureuss	unknow	↑ E-selectin, VCAM1, ICAM1 and IL-6 via the activation of TLR4 and NF-κB signaling pathway	Human dermal microvascular endothelial cells
Onset	Foam cell formation	Porphyromonas gingivalis	LPS	unknow	J774 A.1 cells
		Exosomal CagA derived from *Helicobacter pylori*-infected gastric epithelial cells	CagA	↓ Cholesterol efflux transporters by downregulating the expression of transcriptional factors PPARγ and LXRα	Raw264.7 cells, Human normal gastric epithelial cells lines and ApoE–/– mouse
Progression	Vascular calcification	Porphyromonas gingivalis	LPS	↑ The activity of the osteogenic transcription factor Runx2 by activating the ERK signaling pathway	VSMCs and aortic ring culture model
	Necrotic core formation	Porphyromonas gingivalis	LPS and gingipain	Activating the inflammasome and pyroptotic cell death pathways.	Murine and human macrophages
		Escherichia coli	LPS	Enabling LPS to enter the cytoplasm and activate caspase-11	BMDCs, mouse peritoneal resident cells, THP1 macrophages, and HeLa cells
Rupture	Plaque rupture and thrombosis	Porphyromonas gingivalis	unknow	Inducing platelet aggregation	C57BL/6 mouse whole blood or platelet-rich plasma
		Enterotoxigenic Escherichia coli	LPS	↑ Tissue factor, E-selectin, and P-selectin ↓ Thrombomodulin	HUVECs
		Meningococcal	LPS	↑ Platelet-platelet and platelet-leukocyte aggregation	Citrated whole blood or platelet-rich plasma
		Escherichia coli	unknow	Induce DIC in a TLR4-dependent manner	Wild-type or TLR4 knock-out mice

Table 1: ↑Upregulates; ↓: Downregulates.

- Endothelial Dysfunction and Inflammation: Circulating OMVs carrying LPS or virulence factors from specific bacterial strains may trigger downstream signaling pathways upon binding to TLR4 receptors on endothelial cells, such as activation of the NF-κB signaling pathway, promoting the secretion of pro-inflammatory cytokines and adhesion molecules, facilitating immune cell adhesion to the endothelium. Simultaneously, *P. gingivalis* OMVs cleave endothelial intercellular junction proteins, compromising endothelial integrity, and thereby promoting the entry of monocytes and LDL into the intima. Additionally, *P. gingivalis* OMVs and Sepsis-associated bacteria derived-EVs reduce the secretion of vascular protective factors NO and RNase via the MAPK signaling pathway cascade. These sequential changes create an environment conducive to plaque formation.
- Promotion of Foam Cell Formation: OMVs carrying CagA or LPS downregulate the expression of transcription factors PPARγ and LXRα, inhibiting the transcription of cholesterol efflux transport proteins. This leads to macrophages engulfing modified lipoproteins beyond cholesterol efflux capacity, resulting in intracellular cholesterol ester accumulation and the formation of macrophage-derived foam cells.
- Enhancement of Lipid Core Expansion and Vascular Calcification: *P. gingivalis* and *E.coli* OMVs can be engulfed into the cytoplasm by the endocytic system, and after degradation of the OMV membrane, exposed LPS can directly bind to casepase11 to induce pyroptosis, exacerbating local inflammation by releasing cellular contents from pyroptotic cells and further promoting pyroptosis of surrounding cells. When the clearance capacity of engulfing cells is overwhelmed by dead cells, lipid necrotic cores form. Moreover, *P. gingivalis* OMVs can activate the ERK1/2 signaling pathway, thereby upregulating the osteogenic transcription factor Runx2 in VSMCs, promoting VSMC differentiation into osteoblast-like cells and mineralization, leading to vascular calcification. The enlargement of the lipid core and decreased vascular elasticity further exacerbate plaque instability and the risk of rupture.
- Facilitation of Platelet Aggregation and Thrombus Formation: Endothelial barrier damage may expose underlying connective tissue that could induce platelet activation. Additionally, OMVs derived from Enterotoxigenic *Escherichia coli* can induce endothelial production of procoagulant mediators, promoting platelet activation and aggregation. By affecting inflammatory and coagulation cascades, OMVs-mediated hypercoagulability may lead to localized vascular thrombosis, increasing the risk of cardiovascular adverse events.

### 3.1. BEVs involved in atherosclerosis initiation

#### 3.1.1. Endothelial dysfunction

Endothelial dysfunction is a maladaptive alteration in various functions of vascular endothelial cells, characterized by impaired endothelium-dependent vasodilation, heightened oxidative stress, and increased endothelial permeability. This condition, which is perceived as the preliminary step in atherosclerosis development, is triggered by multiple risk factors such as hypercholesterolemia, oxygen free radicals and hypertension.^48–50^ Several *Helicobacter pylori* virulence-associated proteins, including CagA and VacA, have been repeatedly detected in OMVs.^51,52^ A prospective cohort research found that individuals with CagA-positive *H. pylori* infection had higher levels of endothelin-1 and other endothelial dysfunction biomarkers, suggesting an increased risk of endothelial dysfunction.^53^ This conclusion was further corroborated by Wang et al., who showed that intragastric administration of *H. pylori* OMVs accelerates atherosclerotic plaques formation, further studies found that *H. pylori* OMVs trigger endothelial dysfunction via activation of the reactive oxygen species (ROS)/NF-κB signaling pathway, both CagA and LPS of *H. pylori* OMVs appear to implicated in these processes to some degree.^54^ Studies also indicate that the virulence factor VagA significantly contributes to endothelial dysfunction.^55^ Consequently, specific components of OMVs that significantly contribute to endothelial dysfunction should be identified in subsequent research efforts.

Individuals suffering from periodontitis exhibit an elevated risk of cardiovascular and cerebrovascular diseases, including myocardial infarction and ischemic strokes.^56^ *Porphyromonas gingivalis* is a key pathogen responsible for oral dysbiosis and periodontitis.^57^ Gingipains, a group of cysteine proteases on the cell surface of *P. gingivalis*, play a role in the cleavage of peptides and peptide bonds.^58^ The researchers have identified that OMVs containing gingipains weaken cell-to-cell contacts by cleaving endothelial intercellular junction proteins, including CD31. This disruption impairs the normal functioning of the endothelial barrier and increases vascular permeability.^59^ These alterations facilitate the transendothelial migration of immune cells into the arterial intima and induce vascular inflammation, thereby escalating the risk of cardiovascular diseases. In a state of normal physiological conditions, vascular endothelial cells have the ability to generate nitric oxide, a protective molecule that facilitates vasodilation and maintains vascular homeostasis. Endothelial-type nitric oxide synthase (eNOS) is primarily responsible for nitric oxide expression in endothelial cells.^60^ A recent study revealed that *P. gingivalis* OMVs possess the capability to hinder the expression of eNOS in both human umbilical vein endothelial cells (HUVECs) and mouse aortic endothelium, which may lead to reduced NO secretion and impaired endothelial function.^61^ *Klebsiella pneumoniae* is a Gram-negative bacterium commonly found in the human gastrointestinal tract. Recent studies have discovered that K.pn OMVs significantly impair acetylcholine-induced endothelium-dependent relaxation and elevate superoxide anion production in endothelial cells *in vivo*, which leads to severe endothelial dysfunction and elevated blood pressure. Further investigation revealed that *Klebsiella pneumoniae* OMVs reduce the levels of SIRT1 and p-eNOS in endothelial cells.^62^ In conclusion, OMVs seriously interfere with the vascular endothelium’s ability to maintain homeostasis, raising the risk of ASCVD.

#### 3.1.2. Imbalance of inflammatory mediators in vascular endothelium

The imbalance between pro-resolving and pro-inflammatory mediators leading to a chronic inflammatory milieu in the arterial wall is a key factor in the development of atherosclerosis.^63^ The most potent immunostimulatory component found in OMVs is LPS. As a ligand for Toll-like receptor 4, LPS induces inflammatory responses that can damage host tissues.^64^ In comparison to free LPS, OMVs have been shown to be more effective inducers of atherogenic inflammatory responses.^65–67^ This increased potency may be attributed to the higher antigenicity of OMVs, which have a greater concentration of immunodominant determinants on the vesicles. For instance, the synergistic effect of lipoproteins and LPS leads to severe systemic inflammatory response syndrome.^67^ Some scholars argue that bacterial OMVs should be regarded as a distinct class of pathogen-associated molecular patterns (PAMPs), exerting a more potent effect than free LPS.^68^ Alternatively, OMVs have a greater ability to cross various tissue barriers to reach the site of lesions and to transmembrane translocation compared to free LPS, which exhibits greater hydrophobicity. This enables OMVs to exert a more potent pro-inflammatory effect.^26^

Vascular endothelial cells are one of the major cells in atherosclerotic lesions. When stimulated by LPS, these cells produce inflammatory molecules such as IL-6 and TNF-α.^69^ These substances stimulate the endothelium, allowing white blood cells to be recruited to the site.^48^ Recent studies have shown that treatment with *H. pylori* OMVs enhances IL-6 and TNF-α expression in HUVECs, which can be attributed to the activation of the ROS/NF-κB pathway. When LPS-depleted OMVs are administered, there is a partial normalization of ROS/NF-κB-related protein levels, indicating the partial contribution of LPS in the upregulation of IL-6 and TNF-α.^54^ Similarly, *E.coli* OMVs can trigger an inflammatory cascade in HUVECs by inducing translocation of NF-κB to the nucleus, leading to upregulation of the cytokine IL-6.^70^ Ribonuclease (RNase)1 has been acknowledged as a vessel-protective factor in endothelial cell inflammation that counteracts the adverse effects of extracellular RNA-dependent acute inflammation on plaque progression.^71^ BEVs from sepsis associated pathogens, including *E. coli, Klebsiella pneumoniae* and *Salmonella enterica serovar* Typhimurium, significantly inhibit endothelial RNase1 through a mechanism involving LPS-induced TLR4-IRAK-1 and p38 to promote endothelial cell inflammation and activation.^72^ Compared to bacteria, BEVs are more likely to translocate into the bloodstream due to their nanoscale size and ability to evade host immune responses. These vesicles transport toxins that engage both innate and adaptive immune systems, leading to prolonged chronic inflammatory pathology.^17^ It is conceivable that chronic bacterial colonizers may continuously release BEVs into the bloodstream without causing bacteremia. Given the difficulty in detecting mild inflammation, BEVs might represent an elusive tactic of bacteria, they circulate in the bloodstream and continuously induce local and systemic chronic low-grade inflammation, ultimately triggering the formation of atherosclerotic plaques.

#### 3.1.3. Endothelial activation and monocyte recruitment

Endothelial cell activation refers to its expression of cell-surface adhesion molecules. Under normal circumstances, vascular endothelial cells are able to resist adhesion by monocytes in the bloodstream. However, the stimulation of proinflammatory cytokines and turbulent flow could result in the excessive expression of vascular cell adhesion molecule-1 (VCAM-1), Intercellular adhesion molecule-1 (ICAM-1), and E-selectin,^48^ which are cell surface adhesion molecules that enable leukocyte adhesion and entry into the vascular intima.^50^ Kim et al. uncovered the role of *E. coli* OMVs in enhancing leukocyte adhesion to human microvascular endothelial cells. They demonstrated that the OMVs activate NF-κB, leading to an upregulation of ICAM-1, E-selectin and VCAM-1 expression. LPS was identified as the primary component of OMVs involved in this process.^66^ In addition, *P. gingivalis*, especially its OMVs, promote the expression of Chemoattractant proteins like CXCL1, CXCL2 and CXCL8, which significantly drive the adhesion of monocytes to the HUVEC monolayer.^65^ Similarly, EVs from Gram-positive bacteria can also induce endothelial activation. *Staphylococcus aureus* EVs enhance monocyte adherence to human dermal microvascular endothelial cells (HDMECs) by raising the expression of E-selectin, VCAM1, and ICAM1, as well as IL-6. Notably, HDMECs responded more rapidly and intensely to Staphylococcus aureus-derived EVs than to extracts of Staphylococcus aureus at equivalent protein concentrations.^73^

### 3.2. BEVs involved in atherosclerosis onset

#### 3.2.1. Foam cell formation

Foam cells, which are a key component of the characteristic fatty streaks observed in early-stage atherosclerosis, are formed when monocyte-derived macrophages phagocytose surplus accumulated lipoproteins.^74^ Qi et al. provided evidence that the introduction of *P. gingivalis* OMVs to a mouse macrophage cell line (J774A.1) cultured with LDL induced the formation of foam cells, thereby facilitating the progression of atherosclerosis. The authors speculated that this process was likely mediated, at least in part, by LPS, as OMVs, boiled bacteria, intact bacteria, and purified LPS all stimulated foam cell formation.^75^ In addition, EVs released by gastric epithelial cells of patients with *H. pylori* infection also carry the virulence factor CagA. These EVs inhibit the transcription of cholesterol efflux transporter proteins (including ABCA1, ABCG1 and SR-BI) by downregulating the expression of transcriptional factors PPARγ and LXRα, leading to the generation of macrophage foam cells and promoting the deterioration and instability of plaques.^76^ Although the products of *H. pylori* OMVs can enter circulation via host exosomes, leading to extra-gastric diseases,^77^ the mechanisms by which *H. pylori* and its products traverse the epithelial barrier into the bloodstream remain to be elucidated. Notably, some scholars have identified the presence of OMVs in the circulation.^78^ Therefore, it is essential to discuss whether OMVs themselves, in addition to EVs derived from H. pylori-infected cells, can cross the gastric epithelial barrier and directly impact atherosclerosis.

### 3.3. BEVs involved in atherosclerosis progression

#### 3.3.1. Vascular calcification

Vascular calcification, defined as the deposition of calcium minerals within the arterial wall, is associated with an increased risk of atherosclerotic plaque rupture when it occurs in the intimal layer, which is a hallmark of advanced atherosclerosis.^79^ The mechanism of vascular calcification is thought to be a regulated process similar to mineralization in bone tissue, which is mainly driven by vascular smooth muscle cells (VSMCs). Stimulated by a variety of pro-calcification factors, VSMCs differentiate into osteoblast-like cells expressing the key osteoblast transcription factor Runx2 and other bone-associated proteins.^80^ These phenotypically transformed VSMCs produce matrix vesicles that serve as sites for the deposition of minerals such as calcium phosphates, leading to calcification.^81^ Yang et al. found that *P. gingivalis* OMVs increased Runx2 expression by activating the ERK signaling pathway, leading to VSMCs differentiation and calcification. This phenomenon was observed in both in vitro and ex vivo experiments. Interestingly, VSMCs calcification triggered by OMVs was found to be significantly more severe compared to that induced by LPS at the same concentration, suggesting that additional virulence factors contained in OMVs may contribute to this process.^82^ However, further experimental research is needed to elucidate the involvement of BEVs in vascular calcification.

#### 3.3.2. Necrotic core formation

A prominent characteristic of advanced atherosclerotic plaques is the significant presence of dead cells. Pyroptosis is a type of cell death closely associated with inflammasome activation. It exerts a pro-inflammatory effect and fosters the establishment of large necrotic lipid cores that serve as markers of plaque instability.^1^ The contribution of pyroptosis in various cell types, including macrophages, endothelial cells and VSMCs, to atherosclerosis, has been elucidated.^83^ Since macrophages are the primary constitutive cells in atherosclerotic plaques of humans and mice, and NOD-like receptor family pyrin domain-containing 3 (NLRP3) inflammasome is mainly expressed in macrophages,^84–86^ the event of macrophage pyroptosis can be recognized as the principal accelerator of plaque instability. Fleetwood AJ et al. found that stimulation of macrophages by *P. gingivalis* OMVs leads to a shift in macrophage metabolism from oxidative phosphorylation to glycolysis and the secretion of numerous inflammatory mediators, which activate the inflammasome and trigger pyroptosis. As a result, inflammatory mediators and cytoplasmic compounds are released into the extracellular milieu, which perpetuates local inflammation and further amplifies pyroptosis.^87^ Additionally, OMVs from *E. coli* BL21 or *P. aeruginosa* PAK strain could trigger pyroptosis in a wide range of cells, such as THP-1 macrophages, dendritic cells, and HeLa cells, by facilitating the penetration of LPS into the cytoplasm, which subsequently activates caspase-11.^88^ These findings suggest that OMVs may accelerate the progression of atherosclerosis by promoting extensive cellular death, which facilitates the development of necrotic cores.

### 3.4. BEVs involved in plaque rupture

#### 3.4.1. Plaque rupture and thrombosis

When the fibrous cap that covers the necrotic core breaks apart, blood is exposed to thrombotic substances such as tissue factor (TF) within the core. Thus triggering thrombin production and platelet activation and aggregation, leading to the occlusion of arterial blood flow and the initiation of acute ischemic cardiovascular events.^89^ As mentioned above, *P. gingivalis* OMVs loosened the contact between endothelial cells to disrupt the endothelial barrier, this may expose underlying connective tissue that could induce platelet activation. Moreover, *P. gingivalis* OMVs have been found to induce potent platelet aggregation in vitro.^90^ Additionally, treatment with OMVs derived from Enterotoxigenic *E.coli* significantly upregulates TF expression in HUVECs, while reducing the expression of thrombomodulin, indicating the potential of OMVs to activate the coagulation cascade *in vivo*. Interestingly, inhibition of LPS with its inhibitor polymyxin B was insufficient to suppress changes in the expression of these coagulation cascade mediators, indicating the involvement of OMVs components other than LPS in this process.^91^ Furthermore, Meningococcal OMVs and purified meningococcal LPS can enhance platelet-platelet aggregation and platelet-leukocyte conjugate formation, promoting leukocyte aggregation on the vessel walls and the formation of microthrombosis.^92^ In vivo, *E. coli* OMVs activate the coagulation system in a TLR4-dependent manner, though TLR4 is not required for the activation of the coagulation system under physiological conditions. This leads to the development of disseminated intravascular coagulation.^93^ Altogether, these findings highlight the potentially important role of BEVs in atherothrombosis.

## 4. Bacterial extracellular vesicles: preventing or treating atherosclerosis

**Figure 3.**
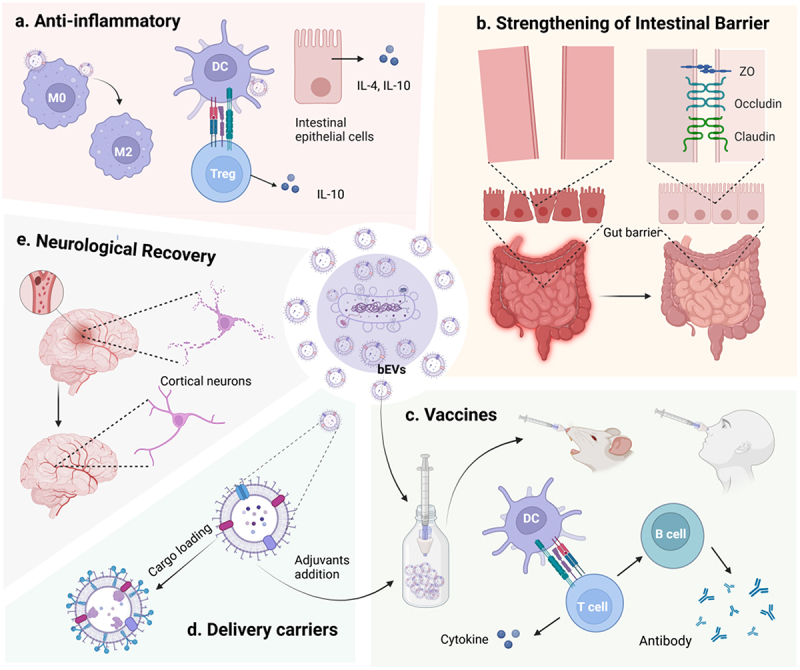
Potential application of bacterial extracellular vesicles in alleviating atherosclerosis.

The beneficial effects of EVs derived from intestinal commensal bacteria and probiotics in treating obesity and insulin resistance by modulating the host’s physiological function have been stated. These diseases, along with atherosclerosis, are categorized as metabolic syndromes with many overlaps in pathogenesis and treatment.^44,94^ Therefore, it is crucial to discuss the beneficial effects of intestinal commensal bacteria and probiotic EVs on atherosclerosis. Table 2 summarizes the findings regarding the beneficial effects of gut commensal and probiotic bacteria-derived EVs on host physiology, primarily involving immunomodulatory functions and improvement of intestinal barrier function. Furthermore, the targeted delivery of drugs and the development of vaccines utilizing engineered bacterial extracellular vesicles are gaining significant attention. In this context, we will discuss their potential for the prevention and treatment of atherosclerosis. The potential effects of BEVs are shown in Figure 3.

**Table 2.** Summary of anti-atherogenic effects of commensal and probiotics bacteria extracellular vesicles.

biological function	Parent bacteria of BEVs	Model	Mechanism
Mitigation of inflammatory response	Lactobacillus plantarums	Human monocytic THP1 cells	↑ Cell-surface markers and cytokines linked to M2 phenotype ↓ M1 macrophage cell-surface markers
	Pediococcus pentosaceus	BMDMs, bone marrow progenitor cells, Mouse models of liver fibrosis, vaccination, peritonitis, colitis, excisional wound	↑ Polarization of M2-like macrophage and myeloid-derived suppressor-like cell differentiation
	Bacteroides fragilis	BMDCs-T Cell Coculture, Colitis mouse model	↑ Regulatory T cells and IL-10 generation through the activation of DCs TLR2
	Akkermansia muciniphila	Human Monocyte-Derived DCs	↓LPS-induced elevation of inflammation-related microRNA ↑ MicroRNA of anti-inflammatory pathways and IL-10
	Faecalibacterium prausnitzii	Human epithelial cell line Caco-2 cells	↓The inflammatory cytokines IL-1，IL-6，IL-17a，IFN-γ ↑ The anti-inflammatory cytokines IL-4，IL-10
	Propionibacterium freudenreichii	Human intestinal epithelial cells HT-29	↓ LPS-induced NF-κB activity and IL-8 secretion
	Akkermansia muciniphila	Colon epithelial cell line CT26 and inflammatory bowel disease mouse model	↓ *Escherichia coli* EVs inducing IL-6 production ↓ Epithelial unsteadiness and inflammatory cell infiltration within colon wall.
Reinforcement of gut barrier	Akkermansia muciniphila	Caco-2 cells and diabetic mice	Ameliorate LPS-induced decrease in TJs by activating the AMPK pathway
	Akkermansia muciniphila	Caco-2 cells, HT-29-MTX-E12 cells, CT-26 cells and Mouse model of gut disorder	↑ Beneficial bacteria proliferation via membrane fusion Triggering mucosal IgA response by translocating into Peyer’s patches and activating B cells and DCs↑ TJs and mucus
	*Escherichia coli* Nissle 1917 and ECOR63	Human colonic cells lines *T*-84 and Caco-2 cells	↑ZO-1 and claudin-14 ↓claudin-2
	*Escherichia coli* Nissle 1917 and ECOR12	Human colonic explants	↑IL-22 and the antimicrobial peptide hBD-2 ↓ IL-12, TGF-β, membrane-anchored mucin MUC1
Alleviating atherosclerosis-related diseases	Lactobacillus plantarum YW11	In vitro hypoxia model and in vivo cerebral ischemia model	reduce neuronal apoptosis and improve neurological recovery after stroke via the miR-101a-3p/c-Fos/TGF-β axis

Table 2: ↑Upregulates; ↓: Downregulates.

- Anti-inflammatory Effects: EVs derived from beneficial bacteria facilitate M2 polarization in macrophages, modulate immune cells, and promote the secretion of anti-inflammatory cytokines.
- Strengthening of Intestinal Barrier: EVs from diverse beneficial bacteria strengthen tight junctions between intestinal epithelial cells, preventing leakage of harmful bacterial products, thus reducing arterial wall inflammation and the area of plaque.
- Vaccines: BEVs vaccination with or without adjuvant induces humoral and cellular immune responses in mice and humans, generating antibodies, activated T cells and cytokines that against the parent bacteria. They hold great potential for preventing infection-related cardiovascular diseases.
- Therapeutic Drug Delivery Carrier: BEVs can serve as targeted carriers for drug delivery, like delivering anti-inflammatory drugs to plaques or target delivery antibiotics to sites of harmful bacterial colonization, thereby alleviating the progression of atherosclerosis.
- Neurological Recovery: Probiotic-derived BEVs or engineered BEVs loaded with therapeutic drugs can cross the blood-brain barrier, and inhibit ischemic neuronal apoptosis, thereby promoting neurological recovery after ischemic stroke.

### 4.1. Beneficial effects of extracellular vesicles derived from intestinal commensal bacteria and probiotics

#### 4.1.1. Immunomodulatory and anti-inflammatory responses

Numerous studies have demonstrated the efficacy of immunomodulatory or anti-inflammatory therapies in alleviating or treating atherosclerosis, which mainly involved correcting the imbalance between pro and anti-inflammatory immune cells in the immune microenvironment of plaques.^1,63,95^ Macrophages within plaques exhibit different phenotypes and functions when stimulated by various factors. M1 macrophages are associated with symptomatic and vulnerable plaques, whereas M2 macrophages are more prevalent in stable plaque regions and asymptomatic lesions.^96^ Recent research has indicated that incubation with EVs derived from *Lactobacillus plantarum* promotes the differentiation of human THP-1 monocytes into an anti-inflammatory M2 phenotype and reduces inflammation-related phenomena associated with M1 macrophages.^97^ Similarly, EVs isolated from *Pediococcus pentosaceus*, a human lactic acid commensal bacterium, promoted the polarization of M2-like macrophages and the differentiation of myeloid-derived suppressor cells (MDSC), which played an anti-inflammatory protective role in several acute inflammation models, including liver fibrosis, vaccination, peritonitis, colitis, and wound healing.^98^ Moreover, Shen et al. observed that treating dendritic cells (DCs) with OMVs containing polysaccharide A(PSA) induces the generation of tolerogenic DCs that improve regulatory T cell (Treg) function, in a process that requires DNA-Damage-Inducible protein (Gadd45α) for PSA-mediated TLR2 signaling in DCs and ultimately provide protection against inflammatory diseases.^99^ Similarly, *Akkermansia muciniphila* and its OMVs can induce tolerogenic DCs that produce anti-inflammatory IL-10 by regulating the expression of microRNAs associated with inflammatory and anti-inflammatory pathways.^100^ These studies highlight the ability of probiotics and commensal bacteria EVs to act as anti-inflammatory and immunomodulatory agents by regulating the differentiation and function of immune cells within plaques. Consequently, they hold promise as therapeutic agents for managing inflammatory disorders like atherosclerosis.

In addition to their inflammatory regulatory effects on immune cells present in atherosclerotic plaques, intestinal microbial EVs also promote the expression of anti-inflammatory factors in intestinal epithelial cells. The risk of ASCVD has been closely linked to inflammatory bowel disease (IBD), and there are significant overlaps in the treatment of these two conditions.^101,102^ A recent study investigated the effect of EVs derived from *Faecalibacterium prausnitzii* on Caco-2 intestinal epithelial cells and found that these cells express lower levels of pro-inflammatory cytokines (e.g. IL-1, IL-6, IL-17a, IFN-γ) and higher levels of anti-inflammatory cytokines (e.g. IL-4 and IL-10) when exposed to the EVs. Notably, the EVs have been shown to be more effective in suppressing inflammation than Faecalibacterium itself.^103^ Similarly, EVs secreted by *P. freudenreichii* attenuated LPS-induced NF-κB activation and IL-8 secretion in intestinal epithelial cells, with the anti-inflammatory effect partially dependent on lamin B.^104^ EVs produced by *A. muciniphila* have also shown potential therapeutic effects on intestinal inflammation. In vitro experiments using colonic epithelial cells (CT26 cell line) demonstrated that prior exposure to *A. muciniphila EVs* counteracted the increased expression of the pro-inflammatory cytokine IL-6 induced by *E.coli* OMVs. *T*reatment with *A. muciniphila* EVs ameliorated the severity of colitis *in vivo*, as evidenced by improved epithelial integrity, reduced inflammatory cell infiltration, and mitigated weight loss and colon length reduction. However, the use of *A. muciniphila* did not result in a protective effect in the condition of a leaky gut.^105^ These studies suggest that in an existing inflammatory environment, the application of live bacteria may disturb the balance of intestinal ecology even further. Therefore, intestinal commensal and probiotic EVs may provide a safer and more effective therapeutic strategy for maintaining intestinal immunity and homeostasis, thereby preventing systemic inflammation resulting from gut dysbiosis and consequent atherosclerosis.

#### 4.1.2. Intestinal barrier strengthening

Maintaining the gut barrier’s integrity is crucial for regulating overall systemic health. Dysfunctions in this barrier have been implicated in the pathogenesis of inflammatory and metabolic diseases.^106^ Specifically, an augmented permeability of the intestinal tract leads to leakage of microbial immunogens (e.g., LPS) and microbial metabolites (e.g. trimethylamine-N-oxide) into the bloodstream, leading to elevated systemic inflammatory levels and the onset of cardiovascular diseases.^106–108^ Intestinal barrier disruption and the associated increased permeability often concur with dysfunction in tight junctions (TJs).^109^ TJs are complex structures integral to the regulation of paracellular permeability. They are primarily composed of several major transmembrane proteins, including occludin, claudins, and zonula occludens (ZO).^110^ Li et al. found an intimate connection between gut microbiota, gut permeability, and cardiovascular vascular system. They proved that *A. muciniphila* can reverse the adverse effects associated with the Western diet-induced increase in intestinal permeability, attenuating atherosclerotic lesions via alleviating inflammation caused by metabolic endotoxemia. However, the underlying mechanism by which gut-residing *A. muciniphila* enhances the expression of epithelial tight junction proteins to improve intestinal barrier function remains to be established.^108^ Chelakkot et al. have extended our understanding of the beneficial impact of *A. muciniphila* on the integrity of gut barrier. Using mice with HFD-induced intestinal dysbiosis and LPS-treated monolayer cultures of Caco-2 cells, they discovered that the decrease in occludin expression was ameliorated by A. muciniphila EVs treatment through the activation of AMPK, thus alleviating LPS-induced barrier impairment in vitro.^111^ According to Wang et al., in mice with intestinal dysbiosis, *A. muciniphila*-derived OMVs stimulate the proliferation of beneficial bacteria by fusing with genus-specific membranes and reduce the relative abundance of harmful bacteria by triggering mucosal IgA response. Additionally, OMVs enhance tight junction expression and mucus secretion. These findings indicate that OMVs maintain intestinal homeostasis by strengthening the intestinal biological barrier, mucosal immune barrier, and intestinal physicochemical barrier.^43^ Additionally, Alvarez et al. have shown that in human intestinal epithelial cell culture monolayers, EVs derived from probiotic *E. coli* strain Nissle 1917 and commensal bacterium ECOR63 could strengthen intestinal epithelial barrier function by enhancing the levels of ZO-1 and claudin-14 and downregulating the expression of the leaky protein claudin-2.^112^ In addition, colonic explants treated with OMVs from probiotic *E. coli* Nissle 1917 or commensal *E. coli* strain ECOR12 exhibited upregulation of IL-22 transcription and the antimicrobial peptide hBD-2, both of which contribute to the integrity of the epithelial barrier. In contrast, bacterial lysates did not exhibit this effect.^95^ In summary, probiotics and gut microbiota-derived EVs have the function of improving intestinal barrier integrity, which can limit the translocation of pro-atherogenic products into circulation. This may be one of the reasons why probiotics exhibit beneficial effects on atherosclerosis.

#### 4.1.3. Regulation of cholesterol metabolism

Hypercholesterolemia is a key risk factor for atherosclerosis. Growing evidence indicates that probiotics can effectively regulate hypercholesterolemia by improving lipid profiles and reducing plasma cholesterol levels, with microbial bile salt hydrolase (BSH) identified as a crucial factor in the cholesterol-lowering effects of probiotics.^113,114^ BSH lowers cholesterol levels in the body by accelerating the conversion of cholesterol into bile acids and, due to the greater hydrophobicity of free bile acids, weakens the formation of cholesterol-stabilized micelles, thereby reducing cholesterol absorption.^114^ Researchers have identified a set of proteins selectively enriched in the BEVs isolated from the cecum of Bacteroides thetaiotaomicron mono-conventionalized germ-free mice using multi-omics analysis, including BSH. Furthermore, intact BEVs degrade bile acids via BSH, indicating the potential beneficial effects of applying probiotic EVs to prevent hypercholesterolemia.^115^ However, the activity of BSH and bile acid metabolism may have multifaceted effects on atherosclerosis and is strain-dependent. Further research is needed to clarify the mechanisms by which probiotic strains and their EVs modify the bile acid metabolism, in order to explore the potential anti-atherosclerotic effects of strategically manipulating the bile acid pool to regulate cholesterol metabolism.^116^ Despite the promising preclinical findings, existing clinical evidence is insufficient to support the use of probiotics as an alternative therapy for improving lipid profiles due to methodological limitations.^117^ The combination of probiotic EVs with other nutritional supplements or lipid-lowering medications could be an intriguing avenue for exploration. This approach has the potential benefit of reducing the dosage of lipid-lowering drugs by enhancing the combined cholesterol-lowering effects.^118,119^

#### 4.1.4. Alleviating atherosclerosis-related diseases

After probiotics enter the human digestive tract, they must compete for adhesion sites and successfully establish colonization. The ultimate effects of probiotics on health are influenced by various host factors, such as the characteristics of baseline microbiota.^120^ In the context of acute ischemic cardiovascular events, the application of probiotics may not produce timely effects. One study demonstrated that EVs derived from edible probiotic strains of *Lactobacillus plantarum* cross the blood-brain barrier, reducing infarct size in a mouse model of ischemic stroke and promoting recovery of neurological function. The underlying mechanism involves LEVs enhancing the expression of miR-101a-3p, which subsequently inhibits the c-Fos/TGF-β axis to limit ischemic neuronal apoptosis.^121^ BEVs retain the effective components of the parent bacteria while overcoming the challenges of colonization, and their flexible and diverse administration methods may offer greater potential for application in acute events related to certain cardiovascular and cerebrovascular diseases.

### 4.2. Application of engineered BEVs in atherosclerosis

#### 4.2.1. As vaccines

In patients with confirmed ASCVD, influenza vaccination has been shown to decrease overall morbidity, mortality, severity of infection, and hospital readmissions among these individuals.^122,123^ Although there is ongoing debate about whether anti-infective therapy can effectively treat atherosclerosis, it is well established that periodontal pathogens and *H. pylori* infections do increase the risk of cardiovascular events. Therefore, screening for these bacterial infections and administering preventive vaccinations in high-risk cardiovascular patients may hold significant public health and clinical value.^56,124–127^ Additionally, active immunization against the outer membrane protein of Enterobacteriaceae improves systemic pro-inflammatory conditions that are associated with the Western diet through apolipoprotein E (apoE)-mediated immune modulation. Such immune stimulation consistently reduced the infiltration of inflammatory cells and increased the fraction of M2 macrophages in atherosclerotic plaques of immunized mice.^128^ ApoE-deficient spontaneously hyperlipidemic mice were intranasally immunized with the outer membrane protein of *P. gingivalis* and cholera toxin as an adjuvant before infection. This immunization significantly reduced the concentration of circulating inflammatory cytokines and the size of atherosclerotic lesions in the aortic sinus of mice intravenously injected with *P. gingivalis* .^129^ These findings might suggest the use of OMVs vaccination as a preventive measure against inflammatory responses and metabolic syndrome resulting from Western-style diets and pathogens.

The unique property of possessing both antigenicity and adjuvanticity positions BEVs as promising candidate vaccine.^130,131^ Currently, several OMV-based vaccines have been developed, and *Neisseria meningitidis* outer membrane vesicle vaccines have also been commercialized.^132^ In a study by Liu et al., the OMVs vaccine against *H. Pylori* was compared to a whole inactivated bacteria vaccine, and it was found that the OMVs vaccine elicited stronger humoral and mucosal immune responses, leading to a significant reduction in *H. pylori* colonization.^133^ Intranasal vaccination with OMVs of *P. gingivalis* has been shown to increase the clearance rate of the pathogen in the oral cavity.^134^ Furthermore, immunization with *P. gingivalis* OMVs significantly reduced levels of MDA-LDL (the most important form of oxidized LDL) in rats and arterial wall thickness, suggesting the potential use of BEVs immunization in preventing or controlling the progression of pathogen-accelerated atherosclerosis.^135^ While tremendous progress has been achieved in the development of BEVs vaccines, evidence of their preventative benefit against atherosclerosis is now restricted to animal models. Therefore, extensive basic research and clinical trials are necessary to evaluate the safety and efficacy of various BEVs vaccines in humans.

#### 4.2.2. As delivery carriers

Although animal studies may show some potential benefits of antibiotics in coronary artery disease, results from human clinical trials do not support the effectiveness of antibiotics in reducing mortality or cardiovascular events in CAD patients.^8,136–138^ The ineffectiveness of antibiotics in these trials does not imply an absence of the association between atherosclerosis and bacterial infection. Rather, it might be attributed to the following reasons: firstly, infection is merely one of many contributing factors and not the indispensable culprit, and antibiotic therapy might be administered too late to achieve significant clinical effects. Secondly, the existence of diverse antibiotic resistance mechanisms in bacteria complicates the complete eradication of those associated with atherosclerotic plaques. Moreover, some studies have proposed the concept of “infection burden”, emphasizing the cumulative effects of chronic infections by multiple organisms on the development of atherosclerosis, rather than the impact of a single pathogen.^139^ This concept may explain the limited efficacy of antibiotic treatment, as viruses do not respond to antibiotics and a single type of antibiotic may not be adequate to target all pathogens contributing to atherosclerosis.

One of the important strategies for bacterial drug resistance is biofilm. It is a highly structured membrane composite consisting of bacteria and the extracellular matrix they secrete, which is firmly attached to a specific matrix.^140^ The formation of biofilms greatly increases bacterial resistance to antibiotics and the host immune system, allowing the development of chronic infections. Recently, some research has identified bacterial biofilms in atherosclerotic plaques by fluorescent microscopy along with fluorescent in situ hybridization. It has been claimed that the existence of these biofilms can accelerate the formation of atherosclerotic plaque by inducing local high-inflammatory environments, as well as raise the risk of plaque instability and rupture.^141–144^ Interestingly, BEVs exhibit a dual effect on biofilm formation. OMVs derived from certain bacteria, such as *H. pylori* and *P. gingivalis*, are believed to promote interactions among bacteria in biofilms, thus contributing to the structural integrity of these formations.^145^ Conversely, OMVs derived from nonpathogenic bacteria contain compounds with antibacterial and anti-biofilm properties that are capable of hindering biofilm formation.^146^ When OMVs are co-administered with antibiotics, the efficacy of the antibiotic against biofilms is enhanced. This enhancement may be attributed to the synergistic effect of OMVs peptidoglycan hydrolase and antibiotics, as well as the ability of OMVs to facilitate antibiotic penetration into biofilms, thereby increasing their efficacy.^147–149^ Given the numerous epidemiological and pathophysiological connections between biofilm formation, chronic inflammation, and atherosclerosis, the use of BEVs loaded with antibiotics or other chemical bactericides is likely to be an effective approach for addressing accelerated atherosclerosis caused by pathogens. Moreover, BEVs offer a potential solution to other bacterial resistance mechanisms, such as drug penetration barriers or alterations in drug target structure. Due to the similarity of their membrane to parent bacteria, BEVs can easily fuse with the outer membrane of target strains, facilitating the delivery of antibiotics into bacterial cells.^150,151^ Advanced techniques have been developed to employ BEVs-coated nano-delivery systems for the targeted delivery of antibiotics into its parent bacteria.^152^ For interested readers, we recommend several review articles that delve into the use of BEVs for antibiotic delivery.^151,153–155^

Given the negative results of most clinical trials on the effects of antibiotic treatment in alleviating atherosclerosis, we suggest that the role of antibiotics in clearing infectious pathogens might be more about reducing pathogen-induced susceptibility to atherosclerosis and lowering the risk of disease onset. For already-formed plaques and chronic low-grade inflammation, effective anti-inflammatory strategies might be more crucial. Currently, the use of widely prescribed anti-inflammatory drugs for atherosclerosis, such as colchicine and methotrexate, is limited due to their conflicting results of safety profile or the absence of clinical benefit.^156–158^ To mitigate the side effects linked to systemic administration, it is imperative to identify more precise targets for cardiovascular inflammation and devise targeted delivery approaches for anti-inflammatory medications to atherosclerotic plaques. Recent studies have underscored the notable impact of anti-inflammatory nanomedicines in modulating inflammation within atherosclerotic plaques.^159–161^ Nanocarriers that package IL-10 can effectively target atherosclerotic plaques in ApoE-deficient mice and release anti-inflammatory cytokines slowly and continuously, resulting in reduced plaque size.^162^ Microbial BEVs possess various PAMPs, which render them advantageous for targeted drug delivery to specific cells, such as neutrophils and macrophages, and being rapidly recognized and internalized. Recently, researchers isolated BEVs from the supernatant of Lactobacillus and constructed the sonosensitizer. Following BEVs administration, PET/CT/NIRF imaging, aortic oil red O staining, and hematoxylin and eosin staining all demonstrated a reduction in plaque area. Further studies indicate that BEVs inhibit atherosclerosis by promoting lipid efflux and macrophage polarization.^163^ Researchers genetically engineered Escherichia coli to extract low-endotoxin OMVs, which were loaded with the neuroprotective agent pioglitazone via electroporation. The OMVs were internalized by neutrophils through the interaction between their LPS components and TLR4 receptors on the neutrophils, thereby enhancing the brain-targeted delivery of pioglitazone for ischemic stroke treatment.^164^ Moreover, through genetic processing, BEVs can load multiple ligands to induce drug deposition at target sites.^165^ It has been observed that the anti-inflammatory effects of Lactobacillus strains’ vesicles in macrophage inflammation models can be improved by optimizing their culture conditions, including pH and stirring speed.^27^ These outcomes imply that BEVs nano-delivery systems hold significant potential for application in inflammatory diseases, offering an alternative approach to mitigate or treat atherosclerosis in the future.

## 5. Challenges and future perspectives

Despite the demonstrated pathogenic effects and therapeutic potential of BEVs in atherosclerosis, current research remains in its early stages. Bridging the gap between basic research and clinical practice will require further exploration and resolution of the following challenges and limitations in future studies:

### 5.1. BEVs research and mass production

Functional analysis and large-scale production of BEVs face several technical challenges. One key issue is contamination from animal-derived EVs during the isolation and purification of BEVs, particularly when using broth culture media such as brain-heart infusion broth. Additionally, there is no consensus on a standardized protocol that covers all pre-processing for BEVs, and current recommendations typically refer to the position papers on BEV research.^166^ Moreover, careful selection of centrifugation speeds is essential to avoid excessive forces that could cause bacterial lysis and release intracellular contents, compromising the purity of the BEVs.^167^ Currently, there is a lack of established technologies and industry-wide quality control standards for large-scale BEV production. Commonly used methods for isolating and purifying BEV populations include differential ultracentrifugation, density gradient centrifugation, ultrafiltration, size exclusion chromatography, and PEG precipitation.^167^ Differential ultracentrifugation remains the most widely used technique, owing to its ability to obtain highly pure BEVs. However, its time-consuming nature and high cost make it unsuitable for industrial-scale production, thereby significantly hinders the clinical translation of BEVs.^168^ Currently, tangential flow filtration is considered the most promising technology for large-scale BEVs production.^169^ With ongoing advancements in industry technology, more effective and cost-efficient methods for large-scale BEV separation and purification are expected to emerge.

Due to the significant impact of the cultivation environment and bacterial strain state on the enrichment and functional activity of BEV components, batch-to-batch consistency in BEV production remains suboptimal.^26^ Therefore, precise control of production conditions is essential to address the stability challenges of BEVs in industrial-scale production. This requires stringent regulation of factors such as bacterial passage number, growth phase, temperature, and pH. Additionally, the morphology, size, concentration, and surface markers of purified BEVs must be characterized prior to functional studies, as this is essential for quality control. Furthermore, a promising alternative to address the challenges of yield, stability, and purity in naturally derived OMVs is the production of bacterial-derived nanovesicles through controlled extrusion.^170^ Another challenge is the incomplete understanding of the physiological concentrations of species-specific BEVs and the effects of different administration routes, which complicates the interpretation of results from in vivo delivery studies. Moving forward, it will be crucial to develop efficient and sensitive techniques for isolating and analyzing BEV subpopulations. In this regard, emerging technologies such as aptamer-based sensors, chemical probe-based chips, and flow cytometry platforms for EV separation and analysis have ushered in a new era for BEV research.^171–173^

In many current studies on BEVs and atherosclerosis, the effective bioactive molecules present on or within BEVs and their specific mechanisms of action remain poorly understood. Further investigation should involve in-depth characterization of various BEVs using metabolomics, proteomics, and (meta)genomic profiling to identify the unique biomolecules and functional effects of different BEVs.^166^ Additionally, genetic manipulation of the parent bacterial strains should be employed to validate the role of BEV-derived effector molecules in disease and elucidate the key targets through which BEVs modulate host immune responses, thereby clarifying the mechanisms by which specific BEVs influence the progression of atherosclerosis. Moreover, while BEVs can be stored at −80°C for research purposes, cost-effective storage methods for industrial production, such as lyophilization and spray drying, hold significant potential.^174^ Given the substantial biological functions of probiotic-derived BEVs, addressing these challenges could pave the way for their development as novel postbiotics in functional foods or health products. Their applications are expected to expand further in the future.

### 5.2. BEVs as diagnostic biomarkers

Given the critical role that BEVs play in the pathogenesis of atherosclerosis, identifying and targeting these vesicles presents promising opportunities for the diagnosis and treatment of ASCVD. A study utilizing a highly efficient and sensitive single BEV detection platform based on nanoflow cytometry identified LPS and lipoteichoic acid as potential BEV biomarkers, highlighting their significant role in assessing intestinal barrier damage.^32^ Notably, pioneering evidence indicates an increased abundance of extracellular vesicles (EVs) containing archaeal DNA, Mycoplasma pneumoniae antigens, and matrix metalloproteinase 9 in the serum of patients with severe myocardial infarction.^175^ Additionally, a study comparing DNA extracted from BEVs isolated from the blood of 198 acute ischemic stroke patients and 200 healthy controls revealed significant differences in blood microbiota composition between the two groups. Specific genera, such as *Ruminococcaceae* and *Prevotella*, were found to be elevated in ischemic stroke patients and were associated with poor prognosis.^176^ This underscores the potential of serum microbiome-derived extracellular vesicles as noninvasive biomarkers for diagnosing or assessing the prognosis of cardiovascular and cerebrovascular diseases.

Analysis of the microbiome composition in stool-derived bacterial extracellular vesicles (stBEVs), using density-based purification and 16S rRNA sequencing, has revealed personalized microbiome profiles. While significant differences exist between stBEV and fecal bacterial compositions, stBEVs provide insights into which bacteria are more likely to produce BEVs in vivo.^177^ In a large-scale case-control study, significant differences in microbiota and BEV metagenomic features were found between patients with IBD and healthy individuals, with BEVs demonstrating superior diagnostic capability for IBD compared to fecal microbiota.^178^ Despite the significant potential of BEVs as diagnostic biomarkers, future research needs to establish high-precision detection platforms for BEVs. These advancements will enable better understanding of the distribution and functions of different BEV subpopulations in the serum, urine, or feces of patients with ASCVD. Such efforts will expand the potential applications of BEVs in personalized and precision medicine.

### 5.3. BEVs as therapeutic targets

Inappropriate use of antibiotics increases mortality in a mouse model of pneumonia caused by multidrug-resistant Klebsiella pneumoniae. The proposed mechanism involves antibiotics promoting the secretion of OMVs from Klebsiella pneumoniae and the presentation of GroEL on the membrane, which facilitates OMV uptake by macrophages, triggering pyroptosis and inflammatory responses. Moreover, the application of OMV-released inhibitors mitigates the increased mortality induced by imipenem without affecting bacterial load.^28^ Currently, the available OMV inhibitors in use are not strain-specific. A critical area for future research is the development of highly selective, effective, and well-tolerated inhibitors for targeting BEVs production or activity.^179^ Such advancements could lay the groundwork for precision medicine in preventing infection-related atherosclerosis.

### 5.4. Safety and efficacy

The administration of live probiotics may increase the risk of gut dysbiosis or bacteremia, particularly in immunocompromised populations.^180^ In contrast, beneficial BEVs present key advantages: they are non-replicative and can elicit beneficial effects similar to their parent bacteria, without the risks associated with viable bacteria entering the bloodstream. However, a potential limitation is the complexity of the components in probiotic EVs. EVs derived from Gram-negative bacteria retain active components such as LPS. While some probiotic LPS may exhibit reduced immune-stimulating activity, safety concerns persist.^181^ Therefore, a systematic evaluation of the bioactive molecules carried by different types of probiotic EVs is essential to assess potential unintended effects. Research has shown that glycine supplementation significantly enhances EVs production from the probiotic *Escherichia coli* strain Nissle 1917, while markedly reducing the endotoxin activity of these EVs.^182^ Consequently, future production processes for BEVs should focus on optimizing in vitro culture conditions to maximize therapeutic efficacy for specific diseases, while minimizing potential side effects.

To ensure the effectiveness of BEVs, further research is needed to investigate their pharmacokinetics and the principles governing the dosing of probiotic EVs. The beneficial impact of a single type of probiotic EVs on host health may be limited. For complex, multifactorial diseases such as atherosclerosis, once the functional effects of different probiotic EVs are clarified, combined formulations of probiotic EVs derived from multiple beneficial bacteria may have a greater positive influence on host health.

## 6. Conclusion

**Figure 4.**
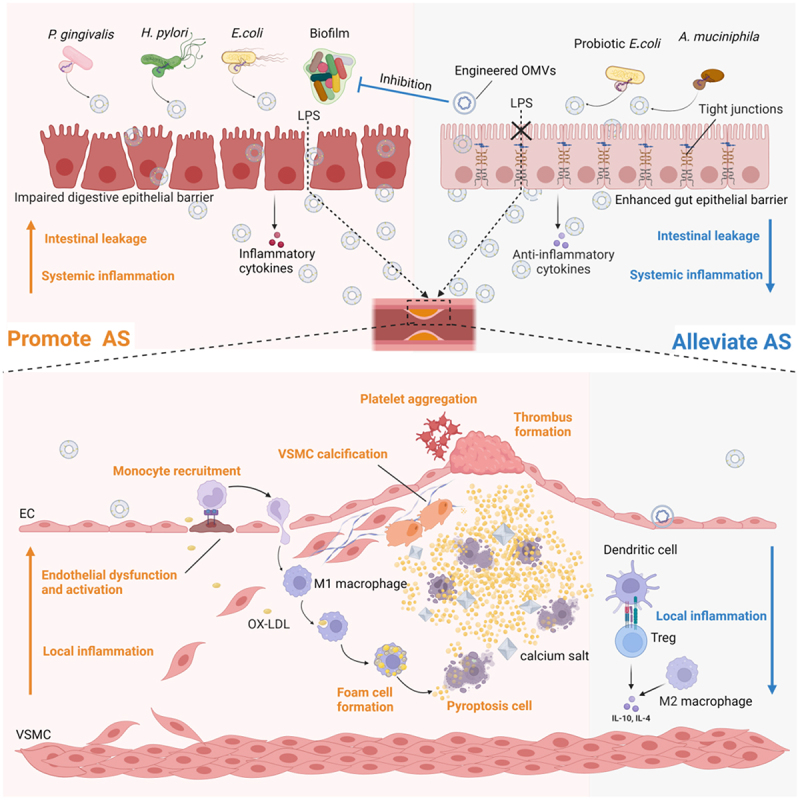
The potential mechanisms by which bacterial extracellular vesicles affect atherosclerosis.

BEVs may play a dual role in mediating both the deleterious and beneficial effects of atherosclerosis by directly or indirectly influencing the local and systemic physiology of the host. The potential mechanisms through which BEVs affect various stages of the atherosclerotic process are illustrated in Figure 4.

**Figure 5.**
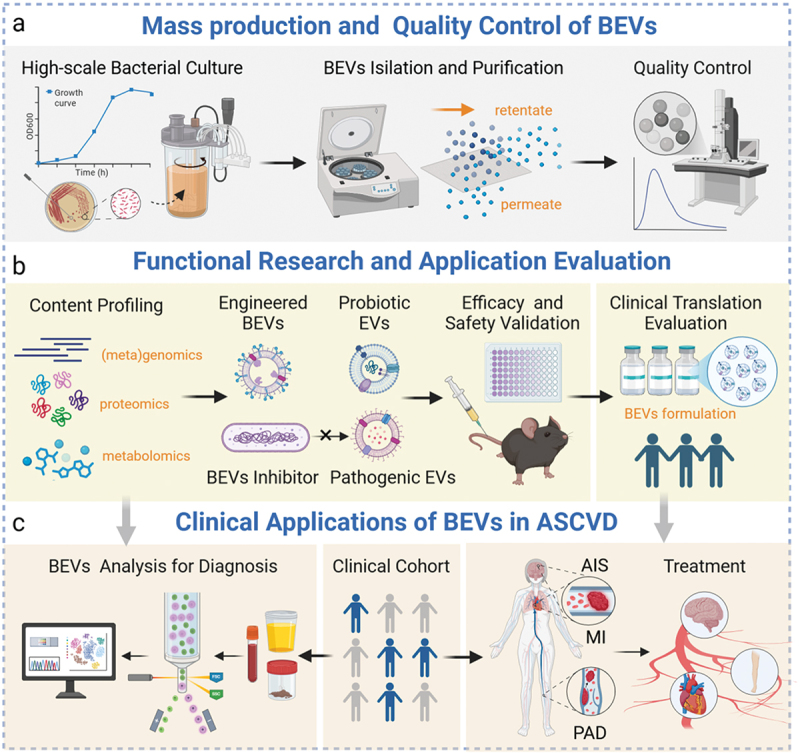
Future research roadmap for the translation of BEVs into clinical applications for ASCVD.

Despite the promising progress in current research, the diverse origins and complex composition of BEVs, along with the limitations of current methodologies, present significant challenges that hinder their application in the clinical diagnosis and treatment of atherosclerosis. Moving forward, further research and the development of innovative technologies are required to elucidate the biological effects of BEVs and establish standardized protocols for their production and research. These advances will pave the way for the translation of BEV-based therapies for atherosclerosis from bench to bedside (Figure 5).

BEVs released by atherogenic microorganisms can traverse the impaired gastrointestinal epithelial barrier and reach the vascular wall, where they promote the formation and progression of atherosclerotic plaques. This is achieved by contributing to various steps in the atherosclerotic process, including endothelial dysfunction and activation and the subsequent accumulation of monocytes and lipids, locally inducing a hyperinflammatory environment, foam cell formation, cell pyroptosis and VSMCs calcification, which enlarges lipid necrotic core, as well as the activation and aggregation of platelets and thrombosis formation. Conversely, gut commensal bacteria and probiotics EVs could mitigate atherosclerosis by preventing gut leakage via increasing the expression of tight junctions that fortify the intestinal barrier, and decrease systemic or local inflammation through interacting with immune cells and intestinal epithelial cells. Engineered BEVs may also prevent atherosclerosis by incorporating them into vaccines that improve the body’s active immunization to remove atherogenic bacteria, or by delivering antibiotics to eradicate biofilms.

(a) Mass Production and Quality Control of BEVs: Large-scale production of BEVs involves culturing bacteria in a stirred bioreactor to optimize yield and quality, with bacterial culture harvested during the bacterial stationary growth phase. BEVs are then isolated and purified in bulk using centrifugation and tangential flow filtration. Quality control assessments are conducted at each step. Characterization of BEVs, including morphology, particle size, and concentration, is performed across batches to ensure batch-to-batch consistency.

- Functional Research and Application Evaluation: Multi-omics techniques are used to analyze the contents of BEVs. Administer probiotic EVs and/or engineered BEVs in vitro and in vivo, or use pathogen-specific BEV inhibitors to suppress the release or activity of pathogenic BEVs in infected mice, to evaluate the safety and efficacy of BEV modulation in improving atherosclerosis. Finally, BEV formulations that have undergone stability, safety, and efficacy evaluations in vitro and in vivo will be assessed for their potential clinical applications.
- Diagnosis and Treatment of ASCVD: Clinical samples, including feces, blood, and urine, are collected for BEV subpopulations analysis using single EV detection technologies. This enables the diagnosis and disease progression assessment of ASCVD. Approved BEV formulations or BEV inhibitors may be used to prevent ASCVD or improve patient outcomes, such as acute ischemic stroke (AIS), myocardial infarction (MI), and peripheral artery disease (PAD).

## Highlight

- Atherosclerosis is a chronic inflammatory disease impacted by various variables, including infection.
- Bacterial extracellular vesicles, which carry various pathogen-associated molecular patterns, translocate to the sites where plaques are prone to form and interact with diverse cells. They participate in several crucial events throughout the plaque formation and advancement process.
- Gut commensal and probiotic bacteria extracellular vesicles alleviate local and systemic inflammation in atherosclerotic events through immunoregulation and intestinal barrier enhancement.
- Engineered bacterial extracellular vesicles have emerged as a feasible approach for developing innovative vaccines and targeted drug delivery, offering potential therapeutic routes for inflammatory diseases associated with infections.

## Nonstandard abbreviations and acronyms

EVs: extracellular vesicles
BEVs: bacterial extracellular vesicles
OMVs: outer membrane vesicles
OX-LDL: oxidized low-density lipoprotein
ASCVD: atherosclerotic cardiovascular disease
CMVs: cytoplasmic membrane vesicles
CagA: cytotoxin-associated gene A
VacA: vacuolar cytotoxin A
ROS: reactive oxygen species
NO: nitric oxide
eNOS: endothelial-type nitric oxide synthase
HUVECs: human dermal microvascular endothelial cells
LPS: lipopolysaccharide
VCAM-1: vascular cell adhesion molecule-1
ICAM-1: Intercellular adhesion molecules 1
HDMECs: human dermal microvascular endothelial cells
VSMCs: vascular smooth muscle cells
NLRP3: NOD-like receptor family pyrin domain-containing 3
TF: tissue factor
MDSC: myeloid-derived suppressor cells
Treg: regulatory T cell
TLR: toll-like receptor
DCs: dendritic cells
apoE: apolipoproteinE
PAMPs: pathogen-related molecular patterns
PSA: Polysaccharide A
IBD: inflammatory bowel disease
